# Inhibitory Potential of Shen-Shuai-Ling Formulation on Renal Interstitial Fibrosis via Upregulation of PLZF

**DOI:** 10.1155/2022/5967804

**Published:** 2022-03-31

**Authors:** Na Song, Haitao Tu, Ying Li, Weijian Xiong, Ling Zhang, Hong Liu, Weisen Ding, Mei Long, Dewei Ren, Jin Zhong

**Affiliations:** ^1^Department of Oncology, Chongqing Hospital of Traditional Chinese Medicine, Chongqing 400021, China; ^2^Department of Nephrology, The First Affiliated Hospital of Guangzhou University of Chinese Medicine, Guangzhou 510405, China; ^3^Department of Nephrology, Chongqing Hospital of Traditional Chinese Medicine, Chongqing 400021, China

## Abstract

**Background:**

Renal interstitial fibrosis (RIF) is an important cause of kidney disease, which seriously affects people's health. As a traditional Chinese medicine, Shen-Shuai-Ling Formulation (SSLF) has obvious kidney function. However, the therapeutic effect of SSLF on RIF and its molecular mechanism are still unclear.

**Methods:**

First, the potential targets and pathways of SSLF for RIF were predicted by network pharmacology, and then, the binding of luteolin and target protein to SSLF was verified by molecular docking and Co-IP experiments. Finally, the effects of SSLF and luteolin on PLZF and (Pro) renin receptor (PRR) were verified by western blot and qPCR experiments. Angiotensin (Ang)-1, Ang-2, and transforming growth factor-*β* (TGF-*β*) were the indexes of renal interstitial fibrosis.

**Results:**

Through the drug-active component-target network diagram, we found that luteolin has the most connections, and promyelocytic leukemia zinc finger (PLZF) is the target protein. GO analysis and KEGG pathway analysis of targets were performed using Cytoscape ClueGO. Molecular docking experiments and Co-IP are used to prove that luteolin and PLZF can be combined. Western blot and qPCR results showed that both SSLF and luteolin significantly upregulated the expression of PLZF and decreased the levels of PRR, Ang-1, Ang-2, and TGF-*β*. The overexpression of PLZF decreased the expression of PRR, the knockdown of PLZF increased the expression of PRR, and the overexpression of PRR decreased the expression of Ang-1, Ang-2, and TGF-*β*.

**Conclusions:**

SSLF inhibits PRR and renal interstitial fibers by the upregulation of PLZF levels.

## 1. Introduction

Chronic kidney disease (CKD) is a disease in which renal structural or functional abnormalities last longer than 3 months. RIF is a common pathological feature of CKD. RIF is the replacement of normal tissue by fibrous tissue and renal failure following chronic and persistent renal injury. Inhibition of RIF is significant in the treatment of CKD.

Excessive accumulation of extracellular matrix (ECM) in the kidneys is an important cause of RIF [1, 2]. TGF-*β* plays a key role in regulating the upstream factors produced by ECM. Ang-2 is of great significance in regulating TGF-*β* upstream factors. PRR plays an important role in regulating the production of Ang-2 [3]. In 2002, a new member of the PRR upstream of the renin-angiotensin system (RAS) was found to be located primarily on the cell surface and at cell intervals, particularly perinuclear spaces [4, 5]. PRR is involved in the production of Ang-2 mainly through two pathways: proteolytic pathway and nonproteolytic pathway [6, 7].

PLZF is also known as zinc finger and BTB (Broad-complex, Tramtrack, and Bric-a-brac) domain containing 16 (ZBTB16) or zinc finger protein 145 (Zfp145). In humans, the PLZF gene is located at position 23 (11q23) of the long arm of chromosome 11, and the gene expression product is a transcription factor composed of 673 amino acids [8, 9]. It is highly conserved in humans, mice, and rats. PLZF is a transcription factor that can act as both a transcriptional inhibitor and a transcriptional activator, and the mechanism of its activation is not known at present. Meanwhile, it has not been elucidated whether PLZF acts as an activator or as a determinant of repression. It is found that renin-treated HEK293 cells can cause PLZF nuclear translocation, and then, PLZF is recruited into the promoter of the PRR gene, which inhibits PRR transcription.

SSLF is a prescription developed by Xin Zheng, a master of traditional Chinese medicine with more than 60 years of clinical experience [10]. SSLF has apparent effects on improving renal function and delaying the progression of CKD and can significantly relieve clinical symptoms such as constipation and abdominal distension.

To study SSLF's functional mechanism and understand its potential inhibitory role in renal fibrosis, we first predicted the possible targets and pathways of SSLF for renal fibrosis using network pharmacology molecular docking, and then, we used molecular docking and Co-IP experiments to verify the binding of SSLF and its luteolin to target proteins. Finally, western blot and qPCR experiments were used to find the mechanism of SSLF and luteolin in inhibiting renal fibrosis.

## 2. Materials and Methods

### 2.1. Experimental Herbal Formulation

SSLF is composed of Herba Rhubarb (DH), Herba Codonopsis pilosula (DS), Herba Astragalus membranaceus (HQ), Herba Stony dragon (ML), Herba Safflower (HH), Herba Ganoderma lucidum (JLZ), Herba Angelica sinensis (DG), Herba Salvia miltiorrhiza (RS), Herba Epimedium (YYH), and Herba Dandelion (PGY). SSLF used in our research was processed by the Chongqing Institute of Traditional Chinese Medicine (Chongqing, China).

### 2.2. Cell Cultures and Experimental Treatments

Human kidney 2 (HK-2) cells were purchased from the American Type Culture Collection (ATCC) (Manassas, USA). HK-2 cells were cultured in DMEM/F12 medium (Gibco). In addition, ten 6-week-old male SD rats were given 20 mg/kg SSLF intragastrically for 7 days. The animal experiment was approved by the Animal Ethics Committee of the Chongqing Hospital of Traditional Chinese Medicine. Rats were euthanized, and serum was collected on day 8. HK-2 cells were seeded and exposed to drug-containing serum or blank serum for 24 hours. HK-2 cells were treated with 10 ng/ml TGF-*β* and 100 nM renin for 24 hours.

### 2.3. Network Pharmacology-Based Analysis

The active components of SSLF were retrieved from the website http://www.swisstargetprediction.ch/. The genes related to human RIF were selected from the GeneCards (https://www.genecards.org/) disease database. The PPI network was constructed by STRING database (https://string-db.org/), and the drug-component-target network was constructed by Cytoscape 3.6.0. The GO analysis and KEGG pathway analysis were performed on 86 target sites with Cytoscape ClueGO, and the enrichment analysis results were visualized.

### 2.4. Molecular Docking

The 3D structure of luteolin was obtained from the TCMSP (https://tcmspw.com/tcmsp.php), the 3D structure of the key target protein was retrieved from the Protein Data Bank (http://www.rcsb.org/pdb), the receptor protein was hydrogenated by AutoDock 4.2.6 software, the charge was calculated, and the receptor protein and ligand molecules were molecularly docked by AutoDock Vina 1.1.2. The confirmation was obtained by docking, the binding energy was scored, and the best binding energy was obtained for graph analysis. PyMOL was used as a three-dimensional graph to show the interaction between the receptor protein and a small molecule of ligand.

### 2.5. Western Blot Analysis

Cells were collected and added to RIPA lysate including PMSF and proteinase inhibitor cocktail. The supernatant was transferred into a precooled 1.5 mL centrifuge tube and centrifuged at 13000 rpm for 20 min for concentration determination. 30 ug/well of total protein was boiled at 100°C for 10 min, separated by 10% polyacrylamide gel electrophoresis (SPS-PAGE), then transferred to a PDVF membrane, closed with 5% skim milk for 1 h at room temperature, and washed 3 times with TBST, 5 min each time. 3% BSA primary antidilution solution was diluted with 1 : 1000∼2000, incubated at 4°C overnight, and incubated with secondary antidilution solution (1 : 1000) for 1 h on the next day.

### 2.6. Coimmunoprecipitation

Luteolin was labeled with biotin by EZ-LinkTM Biotin-LC-Hydrazide (Thermo Scientific). After 24 h incubation, the cells were collected by centrifugation and precooled RIPA buffer was added. The suspension was centrifuged for 15 min. The protein A agarose beads were added and centrifuged for 15 minutes to remove the protein A agarose beads. Add rabbit anti at 4°C overnight. Protein A agarose beads were added to capture the antigen-antibody complex at 4°C overnight. Agarose beads-antigen antibody complexes were collected by centrifugation. The samples were boiled for 5 min. After boiling, the samples were detected by SDS-PAGE electrophoresis.

### 2.7. qPCR

Total RNA was extracted from the sample using TRIzol reagent (Takara, Japan). cDNA was reverse-transcribed using the PrimeScript TM RT kit (Takara, Japan). The response system and procedures for qRT-PCR are described in the instructions of the TB Green Premix Ex Taq II (Takara, Japan), using the CFX96 Real-Time System (Bio-Rad, USA). The relative expression level of genes was calculated by the 2^−∆∆CT^ algorithm. Primer sequence of each gene is as follows: PLZF-F(GCTACTGTGCAAGGCCAACCA), PLZF-R(GCGGTGGAAGAGGATCTCAAACA), PRR-F(GGTAGGGAAGGCAAACTCAGTG), PRR-R(ATTGAGGGGGAGTGAACTGAGAAC), Ang-1-F(AAGATTGCTTCAGCCAGCGTC), Ang-1-R(AGGTGACTTTGGCTACAAGCATTGT), Ang-2-F(TTCAACCTCGCTGTGGCTGA), Ang-2-R(AACTTTGCACATCACAGGTCCAA), TGF-*β*1-F(AATACAGCAACAATTCCTGGCGA), TGF-*β*1-R(CGCACAACTCCGGTGACATCAAA), GAPDH-F(CTGGGCTACACTGAGCACC), and GAPDH-R(AAGTGGTCGTTGAGGGCAATG).

### 2.8. Statistical Analysis

SPSS 23.0 was used to analyze the data. The mean of two groups of independent samples was compared by *t*-test, and the mean of multiple groups of samples was analyzed by one-way ANOVA (*p* < 0.05). All the experiments were repeated three times independently, and the results are expressed as the mean value ± standard deviation.

## 3. Results

### 3.1. Common Targets and Network Visualization of SSLF and RIF

**Figure 1:**
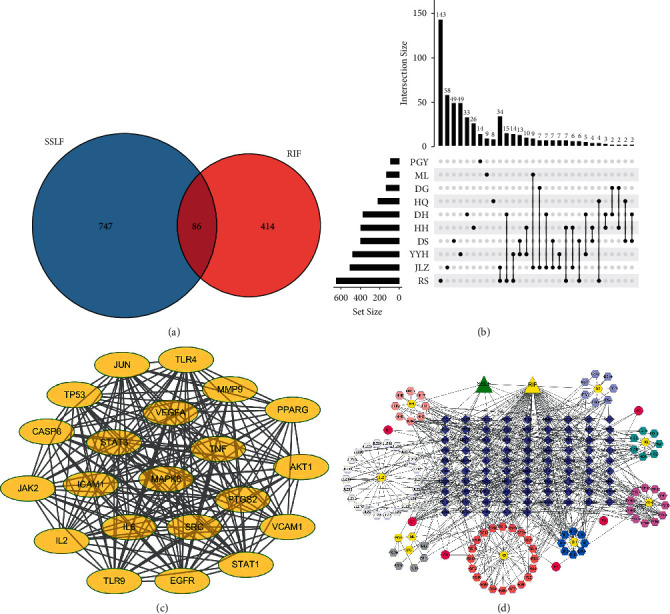
Analysis of key components and core targets of SSLF therapy for RIF based on network pharmacology. (a) Venn diagram of the intersection gene between SSLF and RIF target. (b) Venn diagram of the target intersection gene. (c) Protein-protein interaction network. Edges represent protein-protein interactions. (d) SSLF-compound-target-RIF network. In this network, the blue diamonds represent candidate targets of SSLF against RIF, and the yellow hexagon represents active compounds in SSLF.

In order to study the role of SSLF in RIF, we first predicted 747 targets of SSLF active components in the SwissTargetPrediction database, screened 414 targets of RIF-related genes in the GeneCards database, and obtained 86 intersection genes by intersection analysis (Figure 1(a)). Then we use UpSet plot to visualize the intersection gene between the components of SSLF (Figure 1(b)). We use the STRING database to obtain the protein-interaction network and put the network into the Cytoscape 3.6 to obtain the visual protein interaction network diagram (Figure 1(c)). The network diagram shows that STAT3, VEGFA, and AKT1 are in the central position. Finally, we use Cytoscape 3.6 to analyze SSLF and its components and targets and draw a drug-active component-target network diagram. We find that luteolin has the most connections, and PLZF is the target protein (Figure 1(d)).

### 3.2. GO and KEGG Pathway Enrichment Analyses

**Figure 2:**
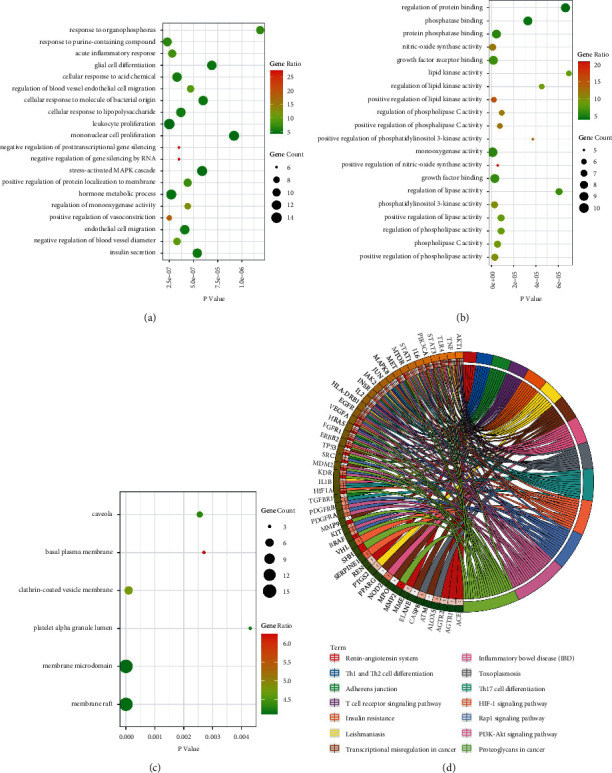
GO and KEGG pathway enrichment analyses of SSLF for RIF. (a–c) The bubble graph shows biological process, cellular component, molecular function, and the intersection gene GO analysis. The left *Y*-axis is the name of the GO path, and the abscissa is *p* value. The larger the circle is, the greater the number of genes compared to this path. The darker the color is, the higher the proportion of genes compared to this path. (d) KEGG pathway enrichment analysis circle diagram. In the outermost layer of the circle, signaling pathway names are displayed in the right side and gene names are displayed in the left side. The inner ring on the left indicates the significant *p* value of gene corresponding pathway.

In order to explore the function of SSLF and the role of potential targets in signaling pathways, we used Cytoscape ClueGO to analyze 86 targets by GO and KEGG pathway and visualized the enrichment analysis results. The GO enrichment analysis shows that, in biological process (BP), it is related to acute inflammatory response, hormone metabolic process, positive regulation of protein localization to the membrane, and negative regulation of gene silencing by RNA (Figure 2(a)). In molecular function (MF), it is associated with the regulation of protein binding, protein phosphatase binding, and growth factor receptor binding (Figure 2(b)). In cellular component (CC), it may be associated with membrane microdomain, clathrin-coated vesicle membrane, and basal plasma membrane (Figure 2(c)). Analysis of the KEGG pathway revealed that 86 potential targets of SSLF for renal fibrosis were primarily associated with the PI3K-Akt signaling pathway, the Rap1 signaling pathway, and the HIF-1 signaling pathway (Figure 2(d)).

### 3.3. Interaction between Luteolin and Target Protein

**Figure 3:**
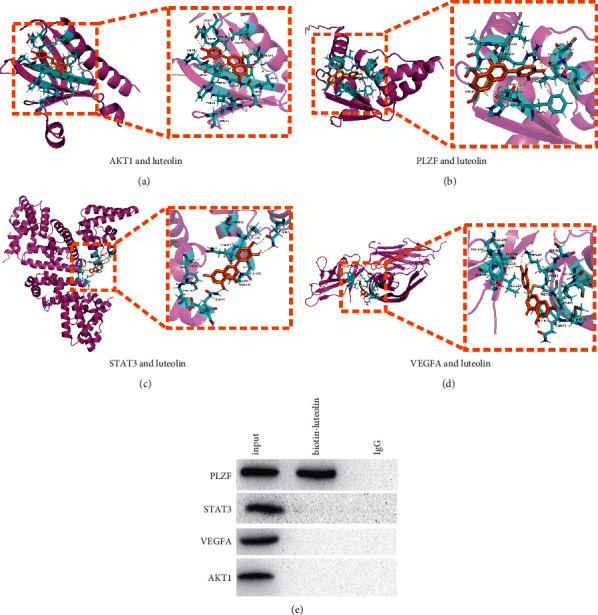
Luteolin and core targets for molecular docking. (a–d) The molecular docking of AKT1, PLZF, STAT3, VEGFA, and luteolin. The yellow line represents the hydrogen bonds; the blue sticks represent amino acid residues. (e) Coimmunoprecipitation assay shows PLZF-luteolin interactions in HK-2 cells.

In order to investigate the effect of luteolin in SSLF on PLZF and core target protein, we first select the key component luteolin and core target genes AKT1 (PDB ID : 1UNQ), VEGFA (PDB ID : 3V2A), STAT3 (PDB ID : 4ZIA), and PLZF (PDB ID : 1BUO) for molecular docking. The binding energies of luteolin and core target proteins were 5.58 kcal/mol (AKT1), 5.25 kcal/mol (VEGFA), 4.08 kcal/mol (STAT3), and 6.48 kcal/mol (PLZF). The amino acid residues (LEU29, ARG28, ALA47, and LEU84) of luteolin and PLZF form hydrogen bonds. The amino acid residues of luteolin and PLZF (ALA-30, GLY-31, GYS-34, GLU-85, GEN-81, HIS-46, and PHE-45) form hydrophobic interactions (Figures 3(a)–3(d)). To verify the relationship between luteolin and PLZF and its target proteins, we labeled luteolin with biotin and observed whether luteolin could bind to PLZF and other target proteins. The Co-IP results show that luteolin can be combined with PLZF (Figure 3(e)). Therefore, we wondered whether SSLF and luteolin could inhibit renal fibrosis by regulating PLZF.

### 3.4. SSLF and Luteolin Upregulate PLZF Levels in HK-2 Cells

**Figure 4:**
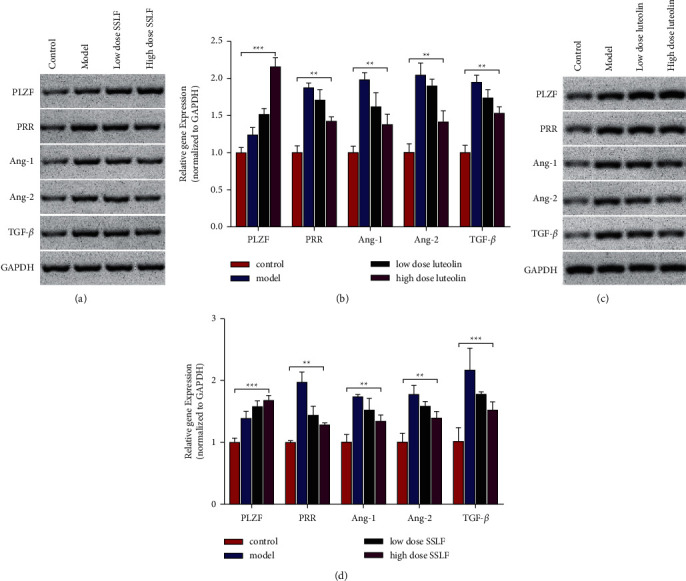
SSLF and luteolin upregulation of PLZF levels. (a, b) Protein and gene expression of PLZF, PRR, Ang-1, Ang-2, and TGF-*β*. Western blot and qPCR analyses were performed after treatment with different concentrations of medicated rat serum containing SSLF. (c, d) Protein and gene expression of PLZF, PRR, Ang-1, Ang-2, and TGF-*β*. Western blot and qPCR analyses were performed after treatment with different concentrations of luteolin (5 *μ*M, 10 *μ*M, and 20 *μ*M). Data are represented as mean ± SD (*n* ≥ 3 experiments). ^*∗*^*p* < 0.05, ^*∗∗*^*p* < 0.01, ^*∗∗∗*^*p* < 0.001, and ^*∗∗∗∗*^*p* < 0.0001 as determined using Student's *t*-test (two groups) or one-way ANOVA, followed by Tukey's test (more than two groups).

In order to verify whether SSLF can regulate PLZF, we first added renin to induce fibrosis of HK-2 cells. The experiment was divided into two groups: the control group and the model group. The model group was divided into three groups. The first group was low-dose SSLF (20% of rat serum concentration) and the second group was medium-dose SSLF (40% of rat serum concentration). The third group was high-dose SSLF (60% of rat serum concentration). The effect of SSLF sum on PLZF was detected by western blot experiment and qPCR experiment. The western blot results showed that SSLF significantly upregulated the expression of PLZF and decreased the levels of PRR, Ang-1, Ang-2, and TGF-*β*. The qPCR results were consistent with the western blot results (Figures 4(a) and 4(b)). Similarly, the results showed that luteolin could upregulate the expression of PLZF and decrease the levels of PRR, Ang-1, Ang-2, and TGF-*β* (Figures 4(c) and 4(d)). Recently, it has been found that renin in HEK293 cells can cause nuclear translocation of PLZF, and then PLZF is recruited to the promoter of the PRR gene, which inhibits PRR transcription. We have confirmed that luteolin can bind to PLZF, and we suspect that luteolin can regulate downstream fibrosis by upregulating PLZF to inhibit PRR expression.

### 3.5. PLZF Reduced the Levels of Ang-1, Ang-2, and TGF-*β* by Inhibiting PRR in HK-2 Cells

**Figure 5:**
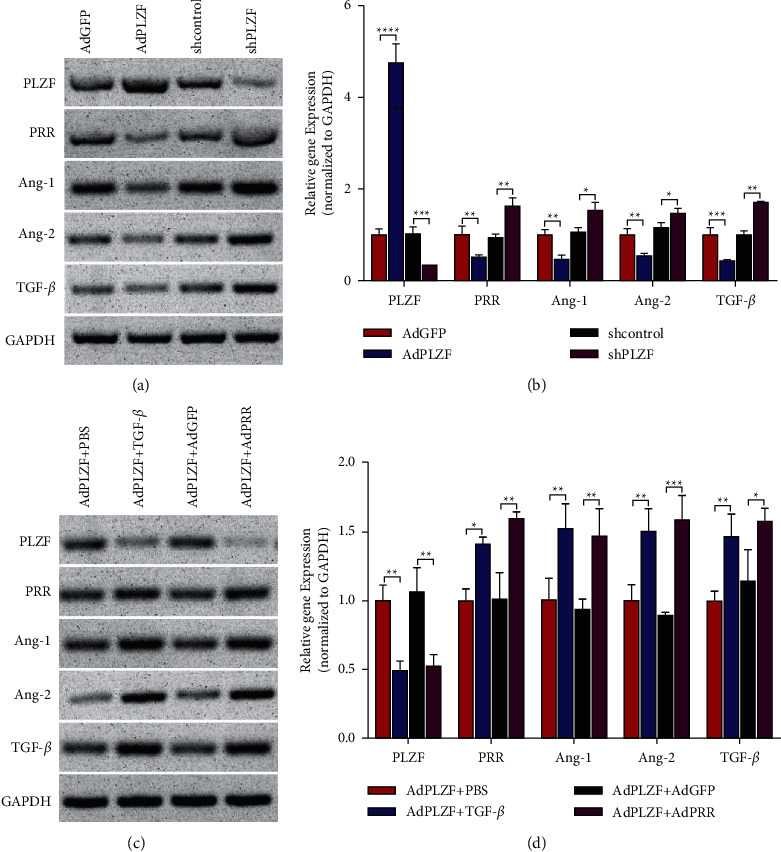
PLZF inhibits PRR expression in HK-2 cells. (a, b) Protein and gene expression of PLZF, PRR, Ang-1, Ang-2, and TGF-*β*. Western blot and qPCR results of overexpression of PLZF and PLZF upon knockdown. (c, d) Protein and gene expression of PLZF, PRR, Ang-1, Ang-2, and TGF-*β*. Western blot and qPCR results of overexpression of PRR. Data are represented as mean ± SD (*n* ≥ 3 experiments). ^*∗*^*p* < 0.05, ^*∗∗*^*p* < 0.01, ^*∗∗∗*^*p* < 0.001, and ^*∗∗∗∗*^*p* < 0.0001 as determined using Student's *t*-test (two groups) or one-way ANOVA, followed by Tukey's test (more than two groups).

In order to study the effect of PLZF on the PRR of a downstream target gene, we first constructed an overexpression cell model and a knockdown model of PLZF. We used western blot and qPCR experiments to verify the effect of PLZF on PRR. The results showed that overexpression of PLZF could decrease the expression level of PRR, knockdown of PLZF could increase the expression level of PRR, and the expression levels of Ang-1, Ang-2, and TGF-*β* were consistent with those of PRR (Figures 5(a) and 5(b)). It was found that renin-treated HEK293 cells resulted in PLZF nuclear translocation, and then PLZF was recruited to the promoter of the PRR gene, which inhibited PRR transcription.

To verify whether PLZF inhibits PRR expression, we induced fibrosis in HK-2 cells with renin and found that the addition of the positive drug TGF-*β* reduced PLZF levels and increased the index of PRR and other indices of renal interstitial fibrosis. In cells overexpressing PLZF, PRR significantly increased the levels of other indices of renal interstitial fibrosis, suggesting that PLZF did inhibit the index levels of renal interstitial fibrosis by inhibiting PRR expression, and qPCR also validated the same conclusion (Figures 5(c) and 5(d)).

## 4. Discussion

CKD is a disease in which renal structural or functional abnormalities last longer than 3 months. At present, CKD is a worldwide public health problem. The prevalence rate increases year by year. With the prolongation of the disease course and the development of the disease, some patients will enter end-stage renal disease (ESRD) [11, 12]. End-stage renal disease patients are in the final stage of chronic renal failure caused by various primary or secondary renal diseases, which are the key diseases endangering human health worldwide. RIF is an important cause of ESRD, in which excessive accumulation and precipitation of ECM in the kidneys lead to the pathogenesis of renal fibrosis. The associated molecular signaling pathways that affect ECM accumulation are complex and include a variety of cytokines as well as hormonal, metabolic, and kinetic factors [13–15]. SSLF contains Herba Rhubarb (DH), Herba Codonopsis pilosula (DS), Herba Astragalus membranaceus (HQ), Herba Stony dragon (ML), Herba Safflower (HH), Herba Ganoderma lucidum (JLZ), Herba Angelica sinensis (DG), Herba Salvia miltiorrhiza (RS), Herba Epimedium (YYH), and Herba Dandelion (PGY). SSLF has apparent effects on improving renal function and delaying the progression of CKD.

To study SSLF's functional mechanism and understand its potential inhibitory role in renal fibrosis, first, we found that STAT3, VEGFA, and AKT1 of the intersection genes of SSLF and RIF are the core proteins in the intersection genes. Using the SSLF-compound-target-RIF network, we found that luteolin, the monomeric drug, has the most connections, and PLZF is the target protein. In order to study the effect of SSLF on PLZF, we used molecular docking and IP experiments to find that the key component of SSLF, luteolin, can bind to PLZF. At present, the mechanism of transcriptional inhibition of PLZF is that PLZF binds to the promoter of the target gene through nine zinc finger structures at the C-terminal, and then its N-terminal BTB/POZ domain recruits nuclear coinhibitory complexes. The physiological functions of PLZF mainly include regulating the differentiation of hematopoietic cells, maintaining the stability of the hematopoietic progenitor cell pool, maintaining the normal development of bone and the renewal of male germ cells, participating in the development of nerve cells, regulating the cell cycle, and participating in cell growth, proliferation, differentiation, and apoptosis. Recent studies have shown that PLZF also participates in immune response and has environmental-dependent anticancer and antitumor effects [16–21].

TGF-*β* is the most effective factor affecting ECM accumulation, which can stimulate ECM production and inhibit ECM degradation. Ang-2 is a member of RAS, and RAS is one of the important pathways closely related to TGF-*β*. The classical RAS is mainly the conversion of renin to Ang-2. Renin is an important hormone secreted by RAS, which can catalyze the conversion of angiotensinogen to Ang-1, and Ang-1 is subsequently cleaved by ACE to Ang-2. It is found that injection of Ang-2 in rats can increase the expression of glomerular TGF-*β*, and blocking the activity of Ang-2 can significantly reduce the expression of TGF-*β*, reduce the formation of ECM, and effectively slow down the progression of disease [22, 23]. It has been reported that PRR plays an important role in regulating the production of Ang-2 [24]. PRR is mainly located on the cell surface and at cell intervals, especially perinuclear spaces. PRR is involved in the production of Ang-2 mainly through two pathways: the proteolytic pathway and the nonproteolytic pathway [25, 26].

To investigate whether SSLF regulates PLZF, we first used renin to induce fibrosis in HK-2 cells. Then, western blot and qPCR experiments showed that SSLF could significantly upregulate the expression of PLZF and decrease the expression of PRR and other indices of renal interstitial fibrosis. Then we also found that luteolin, a key component of SSLF, could upregulate the expression of PLZF and reduce the expression of PRR and other renal interstitial fibrosis markers. The present study found that renin-treated HEK293 cells resulted in nuclear translocation of PLZF, and then PLZF was recruited to the promoter of the PRR gene. We then found that overexpression of PLZF inhibited PRR levels and other indices of renal interstitial fibrosis using western blot and qPCR in a renin-induced model of renal interstitial fibrosis. The knockdown of PLZF resulted in a significant increase in the PRR levels in cells and a significant increase in other indices of renal interstitial fibrosis, and qPCR also validated the same conclusion. This suggests that PLZF can indeed regulate PRR expression. In addition, we found that TGF-*β* reduced the PLZF levels and inhibited PRR and other indices of renal interstitial fibrosis. To verify that PLZF affects renal interstitial fibrosis by regulating PRR, we overexpressed PRR, and western blot and qPCR results showed that, in HK-2 cells, PRR significantly increased the levels of other indices of renal interstitial fibrosis, suggesting that PLZF does inhibit renal interstitial fibrosis by inhibiting PRR expression.

## 5. Conclusions

We first predicted the possible targets and pathways of SSLF for RIF using network pharmacology, and then we used molecular docking and IP experiments to verify the binding of SSLF and its key component, luteolin, to target proteins. Finally, using western blot and qPCR experiments, we found that SSLF and luteolin could upregulate the expression of PLZF and inhibit the level of PRR. In future studies, we will further verify the effect of SSLF and luteolin in regulating renal interstitial fibrosis through PLZF in animal models and collect clinical data to analyze the relationship between the consumption of SSLF and the expression level of PLZF in the body to provide a basis for better clinical applications.

## Data Availability

The data supporting the research are included in this study.

## References

[1] Thallas-Bonke V., Lindschau C., Rizkalla B. (2004). Attenuation of extracellular matrix accumulation in diabetic nephropathy by the advanced glycation end product cross-link breaker ALT-711 via a protein kinase C-*α*−dependent pathway. *Diabetes*.

[2] Inoguchi T., Sonta T., Tsubouchi H. (2003). Protein kinase C-dependent increase in reactive oxygen species (ROS) production in vascular tissues of diabetes: role of vascular NAD(P)H oxidase. *Journal of the American Society of Nephrology: Journal of the American Society of Nephrology*.

[3] Leung J. C. K., Chan L. Y. Y., Saleem M. A., Mathieson P. W., Tang S. C. W., Lai K. N. (2015). Combined blockade of angiotensin II and prorenin receptors ameliorates podocytic apoptosis induced by IgA-activated mesangial cells. *Apoptosis*.

[4] Nguyen G., Delarue F., Burcklé C., Bouzhir L., Giller T., Sraer J.-D. (2002). Pivotal role of the renin/prorenin receptor in angiotensin II production and cellular responses to renin. *Journal of Clinical Investigation*.

[5] Shan Z., Cuadra A. E., Raizada M. K. (2008). Characterization of a functional (pro)renin receptor (PRR) in brain neuron. *The FASEB Journal*.

[6] Nguyen G., Muller D. N. (2009). The biology of the (Pro)Renin receptor. *Journal of the American Society of Nephrology*.

[7] Nabi A. H. M. N., Suzuki F. (2010). Biochemical properties of renin and prorenin binding to the (pro)renin receptor. *Hypertension Research*.

[8] Jin Y., Nenseth H. Z., Saatcioglu F. (2017). Role of PLZF as a tumor suppressor in prostate cancer. *Oncotarget*.

[9] Rohle D., Popovici-Muller J., Palaskas N. (2013). An inhibitor of mutant IDH1 delays growth and promotes differentiation of glioma cells. *Science*.

[10] Liu H., Xiong W., Zheng X. (2016). Academic thought and clinical experience of TCM master ZHENG Xin in treating diabetic nephropathy. *China Journal of Traditional Chinese Medicine and Pharmacy*.

[11] Luyckx V. A., Cherney D. Z. I., Bello A. K. (2020). Preventing CKD in developed countries. *Kidney international reports*.

[12] Zha Y., Qian Q. (2017). Protein nutrition and malnutrition in CKD and ESRD. *Nutrients*.

[13] Farris A. B., Colvin R. B. (2012). Renal interstitial fibrosis. *Current Opinion in Nephrology and Hypertension*.

[14] Shrestha A., Che R.-C., Zhang A.-H. (2019). Role of aldosterone in renal fibrosis. *Advances in Experimental Medicine and Biology*.

[15] Sun Y. B. Y., Qu X., Caruana G., Li J. (2016). The origin of renal fibroblasts/myofibroblasts and the signals that trigger fibrosis. *Differentiation*.

[16] Barna M., Hawe N., Niswander L., Pandolfi P. P. (2000). Plzf regulates limb and axial skeletal patterning. *Nature Genetics*.

[17] Dick J. E., Doulatov S. (2009). The role of PLZF in human myeloid development. *Annals of the New York Academy of Sciences*.

[18] Ching Y.-H., Wilson L. A., Schimenti J. C. (2010). An allele separating skeletal patterning and spermatogonial renewal functions of PLZF. *BMC Developmental Biology*.

[19] Bernardo M. V., Yelo E., Gimeno L., Campillo J. A., Parrado A. (2007). Identification of apoptosis-related PLZF target genes. *Biochemical and Biophysical Research Communications*.

[20] Savage A. K., Constantinides M. G., Han J. (2008). The transcription factor PLZF directs the effector program of the NKT cell lineage. *Immunity*.

[21] Hobbs R. M., Pandolfi P. P. (2010). Shape-shifting and tumor suppression by PLZF. *Oncotarget*.

[22] Vega G., Alarcón S., San Martín R. (2016). The cellular and signalling alterations conducted by TGF-*β* contributing to renal fibrosis. *Cytokine*.

[23] Sutariya B., Jhonsa D., Saraf M. N. (2016). TGF-*β*: the connecting link between nephropathy and fibrosis. *Immunopharmacology and Immunotoxicology*.

[24] Xu Q., Jensen D. D., Peng H., Feng Y. (2016). The critical role of the central nervous system (pro)renin receptor in regulating systemic blood pressure. *Pharmacology & Therapeutics*.

[25] Li Q., Raizada M. K. (2010). Is (pro)renin receptor a multifunctional receptor?. *Hypertension (Dallas, Tex.: 1979)*.

[26] Yang T. (2015). Crosstalk between (Pro)renin receptor and COX-2 in the renal medulla during angiotensin II-induced hypertension. *Current Opinion in Pharmacology*.

